# Case report: Rubella virus-associated cutaneous granuloma in an adult with TAP1 deficiency

**DOI:** 10.3389/fimmu.2024.1366840

**Published:** 2024-04-12

**Authors:** Qiaohui Wang, Huilin Su, Jiande Han, Juhua Yang, Naiyu Lin

**Affiliations:** ^1^ Department of Dermatology and Venereology, The First Affiliated Hospital of Sun Yat-sen University, Guangzhou, Guangdong, China; ^2^ Vision Medicals Co., Ltd., Guangzhou, Guangdong, China

**Keywords:** rubella virus, cutaneous granuloma, TAP1 deficiency, immunodeficiency, diagnosis

## Abstract

Rubella virus-associated granulomas commonly occur in immunocompromised individuals, exhibiting a diverse range of clinical presentations. These manifestations can vary from predominantly superficial cutaneous plaques or nonulcerative nodules to more severe deep ulcerative lesions, often accompanied by extensive necrosis and significant tissue destruction. TAP1 deficiency, an exceedingly rare primary immune-deficiency disorder, presents with severe chronic sino-pulmonary infection and cutaneous granulomas. This report highlights the occurrence of rubella virus-associated cutaneous granulomas in patients with TAP1 deficiency. Notably, the pathogenic mutation responsible for TAP1 deficiency stems from a novel genetic alteration that has not been previously reported. This novel observation holds potential significance for the field of diagnosis and investigative efforts in the context of immunodeficiency disorders.

## 1 Introduction

Cutaneous granulomatosis is a heterogeneous group of diseases, characterized by a skin inflammatory reaction triggered by a wide variety of stimuli, including infections, foreign bodies, malignancy, metabolites, and chemicals. However, virus-related chronic cutaneous granulomas are less common and are often linked to immune deficiencies (1). In recent years, there has been increasing attention and reporting on cutaneous granulomas associated with congenital immune deficiencies (2). Transporters associated with antigen processing (TAPs) play a crucial role in the stability and surface expression of major histocompatibility complex class I molecules in all nucleated cells. Defects in genes related to TAP expression can result in autoimmune deficiency-related disorders. Previous literature has documented cutaneous granulomatous diseases linked to deficiencies in TAP1 and TAP2 (3, 4).This report documents a case of rubella virus-associated cutaneous granuloma associated with a defect in the TAP1 gene, which has not been reported previously and is helpful in improving clinicians’ understanding of these rare diseases.

## 2 Case description

**Figure 1:**
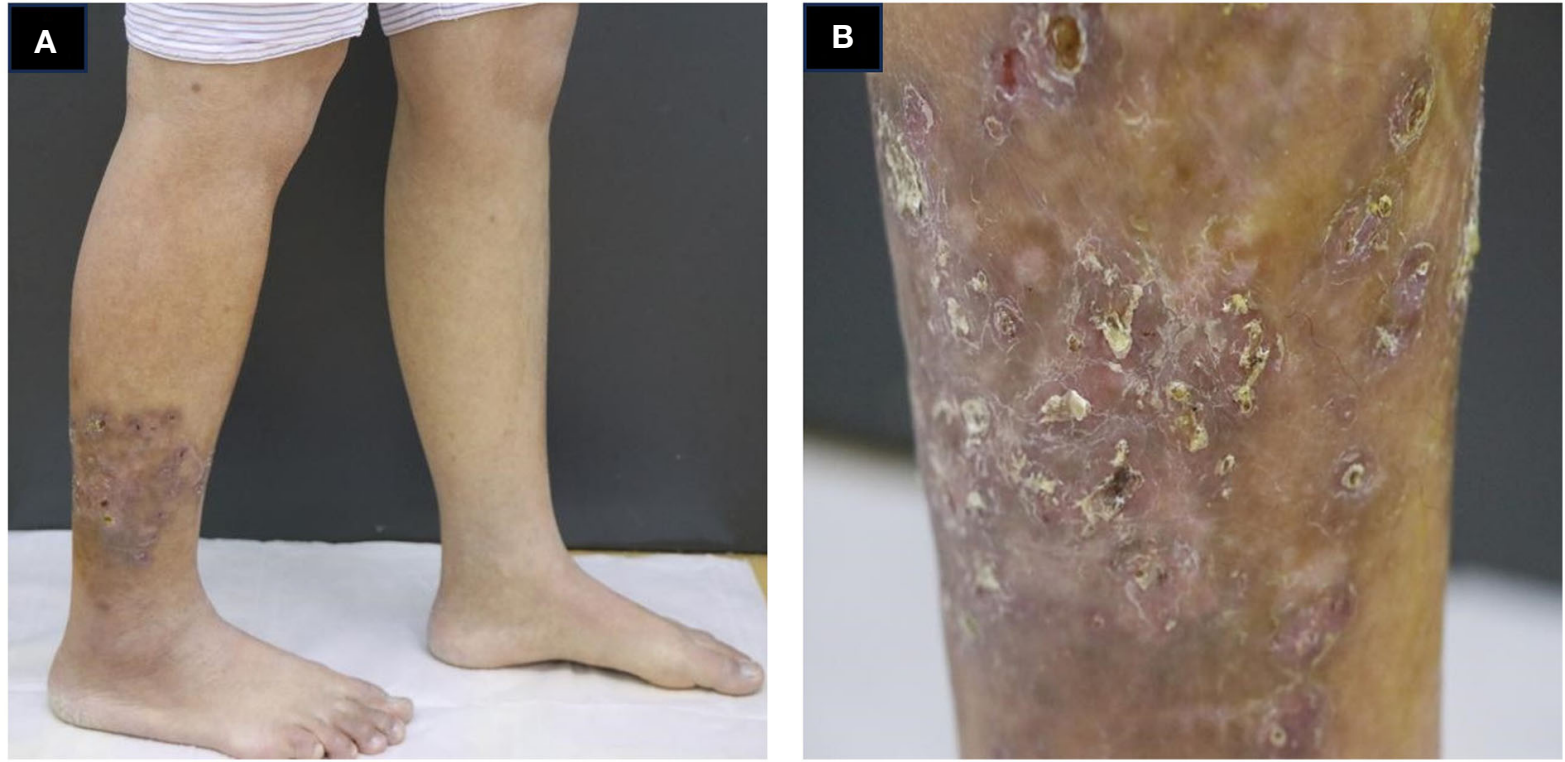
Clinical presentation of the patient. **(A, B)** infiltrated dark red plaques distributed across the right lower extremity, overlaying discrete circular superficial ulcers characterized by a lack of exudation.

**Figure 2:**
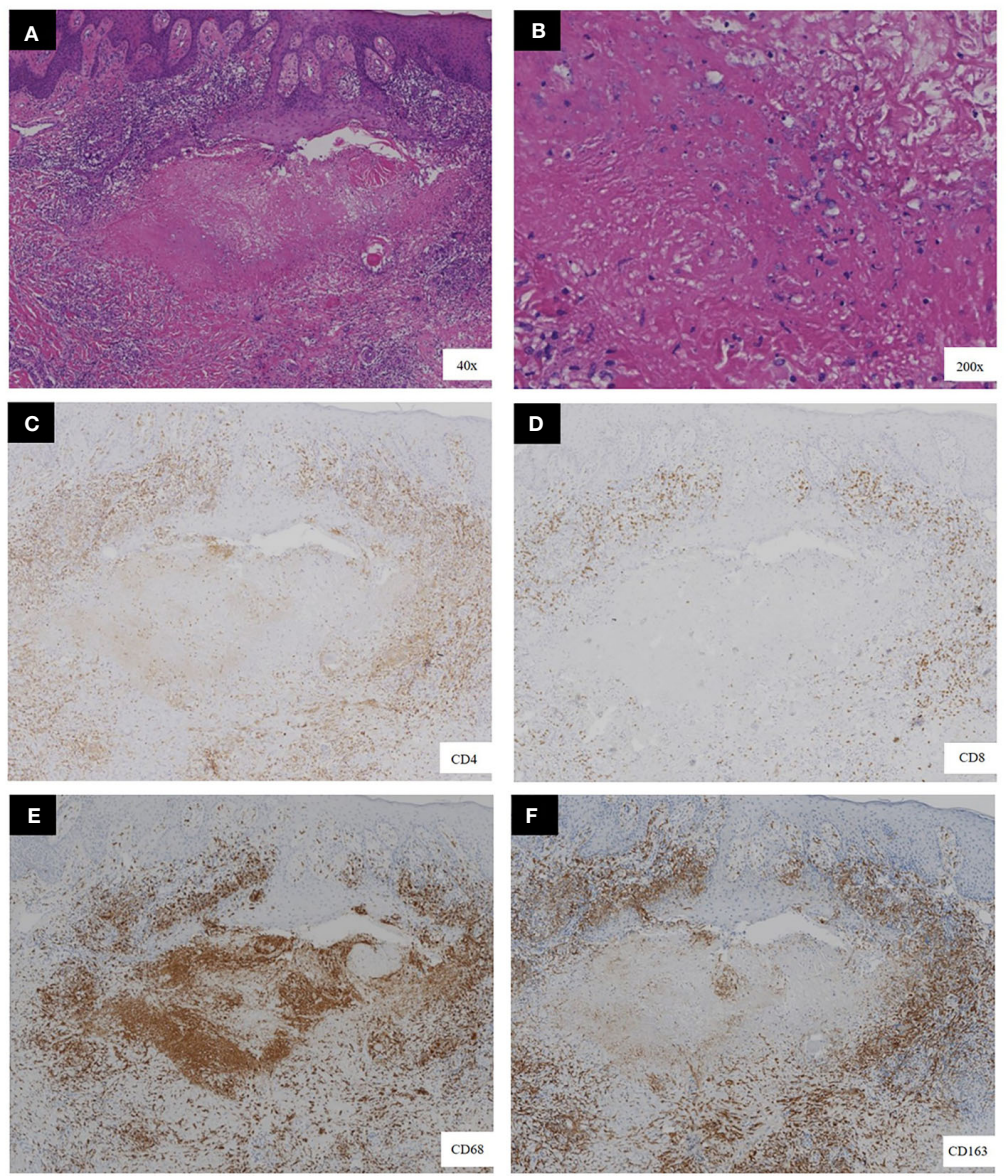
Histopathological features of the biopsy. **(A, B)** the presence of necrotizing granulomas with variable lymphocytic infiltration, as demonstrated by hematoxylin-eosin staining. **(C–F)** Immunohistochemical staining showed a significant proportion of the infiltrating cells located at the granuloma center exhibited CD68 positivity, indicative of macrophages. Surrounding these macrophages were T lymphocytes, notably positive for both CD4 and CD8.[original magnification ×40 in A, ×200 in B, and ×40 for all images in **(C–F)**].

**Figure 3:**
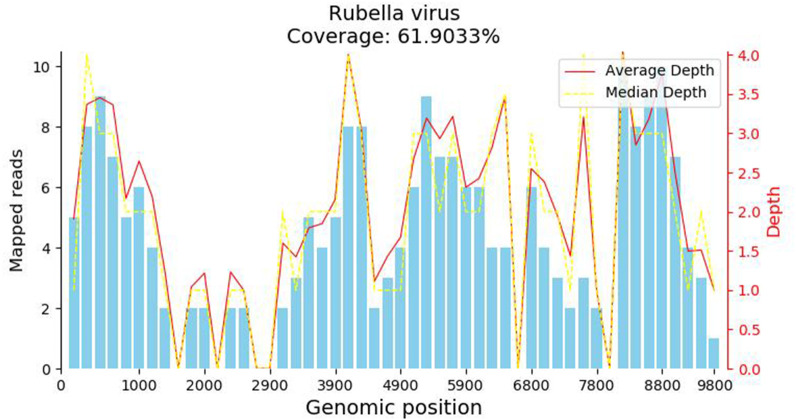
The metagenomic next-generation sequencing (mNGS) test result. The metagenomic next-generation sequencing (mNGS) test identified a total of 368 reads corresponding to rubella virus, with a coverage of 61.9033%.

A 34-year-old female patient presented with a seven-year history of non-painful plaques affecting her right leg, which had recurrently progressed to ulceration and crusting. Upon physical examination, the clinical presentation revealed dark red plaques distributed across the right lower extremity, overlaying discrete circular superficial ulcers characterized by a lack of exudation (**Figure 1**). Notably, no palpable enlargement of nearby superficial lymph nodes was detected. The patient’s medical history and prior diagnostic and therapeutic interventions had already excluded bacterial, fungal, and tubercular etiologies. Despite these investigations, the patient’s condition had continued to deteriorate, prompting consideration of autoimmune diseases as a potential underlying cause, including the possibility of vasculitis. Laboratory analysis of peripheral blood revealed the presence of a specific cellular immune deficiency in the patient, characterized by a decreased count of CD3+ T cells (499.48 cells/µl) and NK cells (69.52 cells/µl) (**Table 1**). To further refine the diagnosis, a skin biopsy was performed, and a portion of the tissue was subjected to metagenomic next-generation sequencing (mNGS) testing. Histopathological examination revealed dense granulomatous inflammation with variable lymphocytic infiltration. The overall granulomatous patterns exhibited a palisaded, sarcoidal, and suppurative nature with caseation (**Figures 2A, B**). Immunohistochemistry results indicated that the predominant infiltrating cells within the granuloma were CD4+/CD8+ positive lymphocytes and CD68+/CD163+ macrophages (**Figures 2C–F**). Various staining techniques, including Gram, periodic acid–Schiff, and acid-fast bacteria stains, as well as tissue cultures for bacteria, fungi, and mycobacteria, yielded negative results. Notably, the tissue mNGS test, conducted on the Vision Medical Center’s Illumina Nextseq 550Dx platform, indicated the presence of Rubella Virus (RuV). The test identified a total of 368 reads corresponding to rubella virus, with a coverage of 61.9033% (**Figure 3**). Due to the limited number of reads, no specific information about the RuV strains could be provided, which was insufficient for assembly and detailed characterization of viral strains.

**Table 1:** Laboratory tests of the patient.

Tests	Results	Reference ranges
Total white blood count (WBC)	4.39 × 10^9^/L	4.00-10.00 × 10^9^/L
Neutrophils count (Neut)	2.94 × 10^9^/L	1.80-6.40 × 10^9^/L
Lymphocyte count (LY)	0.97 × 10^9^/L	1.00-3.30 × 10^9^/L
CD3^+^CD19^-^ T Cell	499.48/ul	603.00-2993.00/ul
CD3^-^CD19^+^ B Cell	530.55/ul	107.00-698.00/ul
CD3^+^CD4^+^ T Cell	351.11/ul	441.00-2156.00/ul
CD3^+^CD8^+^ T Cell	122.35/ul	125.00-1312.00/ul
CD3^-^CD16^+^CD56^+^ NK Cell	69.52/ul	95.00-640.00/ul
IFN gamma Antibodies(Blood)	negative	negative
metagenomic next-generation sequencing (mNGS) (biopsy tissue sample)	Rubella virus	negative
RuV RNA test(Skin biopsy tissue)	positive	negative
RuV RNA test (Blood)	negative	negative
Serum RuV IgM titers	0.05	≤1.2 IU/mL
Serum RuV IgG titers	>500.00	0.00-5.00 IU/mL

Consequently, we initiated additional RuV-related assessments. Upon further inquiry, the patient denied prior MMR (measles, mumps, and rubella) vaccination and any history of prior RuV infection, or any related family history. Strikingly, her RuV IgG titer was significantly elevated (>500 IU/mL), while RuV IgM remained seronegative. Tissue RT-PCR testing for RuV RNA also returned positive results. Considering the patient’s medical history and the corroborative laboratory findings, the diagnosis of RuV-associated granuloma was established.

**Figure 4:**
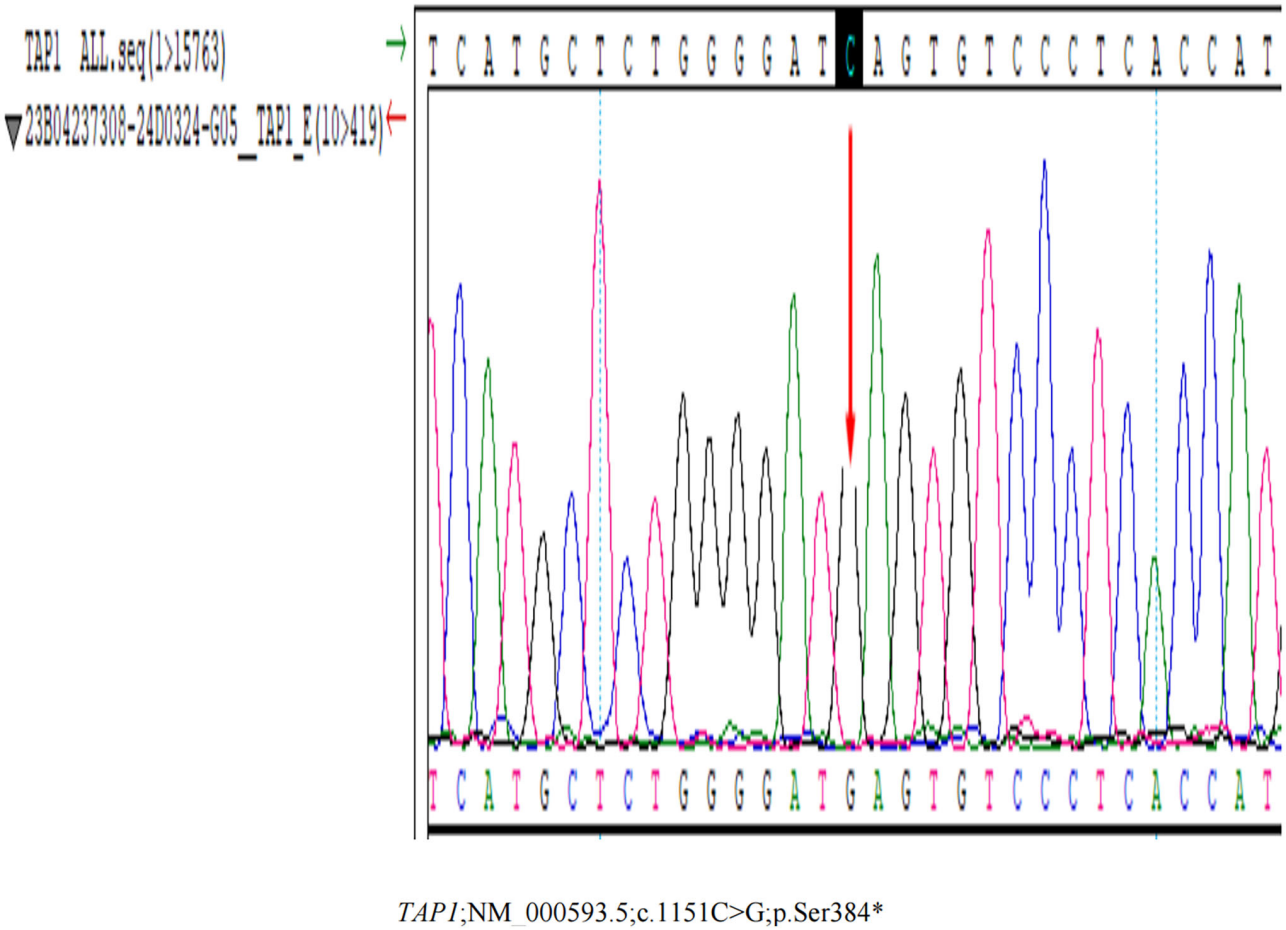
The Whole Exome Sequencing Testing result. The Whole Exome Sequencing Testing reveals a homozygous mutation NM_000593.5:c.1151C>G (p.Ser384*) located in TAP1.

Given the possibility of an underlying immunodeficiency, Whole Exome Sequencing was undertaken, revealing a homozygous mutation NM_000593.5:c.1 151C>G (p.Ser384*) located in TAP1 (**Figure 4**; **Supplementary Data 1**), which was verified by Sanger Sequencing. As of now, there are no reported findings on the pathogenicity of this mutation. However, according to the guidelines set by the American College of Medical Genetics and Genomics (ACMG) (5), this variant is classified as a pathogenic variant, as it meets the criteria of “PVS1+PM2+PM3_Supporting”. In response to the diagnosis of TAP1 deficiency, we conducted comprehensive systemic examinations on the patient, and no other abnormalities were found.

Treatment modalities employed included surgical resection, intralesional injection of interferon α-2b once in 3 days, and topical application of imiquimod 3 times a week. Treatment was well tolerated, and the patient experienced no side effects. Follow-up are currently ongoing, with preliminary indications suggesting surgical resection may be a more effective treatment, as there were no signs of recurrence after 11 months of follow-up.

## 3 Discussion

Rubella virus (RuV) is a single-stranded RNA virus capable of inducing persistent infections that can lead to various associated pathologies (6). Notably, in 2014, RuV-associated cutaneous granulomas afflicting patients diagnosed with primary immunodeficiency diseases (PID) was first reported (7). The severity of RuV-associated granulomatous inflammation exhibits a wide spectrum, ranging from a limited number of superficial cutaneous plaques or nonulcerated nodules to more severe presentations featuring deep ulcerated lesions accompanied by necrosis (8). In those patients, serum samples tested negative for RuV-IgM. Conversely, RuV-IgG titers were significantly elevated, with values falling within a range of 580-1623 IU/mL. These elevated IgG titers far exceeded those typically observed in immunologically intact individuals following Rubella vaccination, which typically average around 40 IU/mL (9, 10). In our case, this patient displayed characteristic lower extremity cutaneous manifestations and marked elevation in RuV IgG levels, aligning with patterns observed in previous reports (9).

RuV-associated granulomas are predominantly sarcoidal epithelioid type consisting of M2 type (CD68+/CD206+ and CD68+/CD163+) macrophages harboring RuV antigen at the granuloma center surrounded by lymphocytes. Four distinct granuloma patterns can be classified:M-type RuV-associated granuloma, M(n)-type RuV-associated granuloma, N-type RuV-associated granuloma and DNI-type RuV-associated granuloma (11). The histopathological examination of our case reveals a distinctive amalgamation of necrotizing and non-necrotizing granulomas within a single biopsy specimen. Notably, this histological pattern is marked by the presence of CD68+/CD168+ M2 macrophages, encircling acellular necrotic centers. These necrotic centers exhibit dense infiltration by CD3+ T cells and, in some instances, the presence of neutrophils is noted, positioned in the interstitial space between the ring of macrophages and the necrotic centers. This histological configuration closely aligns with the characteristic features associated with M(n)-type Rubella virus (RuV)-associated granulomas. MHC class I molecules are expressed by all nucleated cells, have roles in intracellular peptide antigen processing and presentation, thymocyte development in thymus, regulation of the activities of natural killer (NK) and γδ T cells. Major histocompatibility complex (MHC) class I deficiency syndrome, also known as bare lymphocyte syndrome(BLS) type II, due to TAP1, TAP2 and Tapasin deficiency, is a rare autosomal recessively inherited primary immunodeficiency disease (12). Among individuals with TAP1 deficiency, a significant proportion manifests severe, chronic bacterial infections affecting the respiratory tract and/or experiences the development of severe, chronic cutaneous granulomatous lesions mimicking Wegener’s granulomatosis (13). Interestingly, viral infections are not prominent in TAP1 deficient patients. It is presumed that TAP-independent immune responses provide adequate protection against viral infection (14), or alternatively, immune tolerance may develop over time in response to these pathogens. Nonetheless, the patient we have presented in this report was diagnosed with TAP1 deficiency and concurrently experienced the complicating factor of RuV-induced cutaneous granuloma, thus highlighting a unique clinical scenario. This case provides valuable diagnostic insights. Molecular diagnostic techniques such as RT-PCR or mNGS applied to skin biopsy tissues can aid in confirming the presence of these infections. It is worth noting that using secretions for RT-PCR or mNGS is not recommended due to their lower diagnostic yield compared to tissue samples. Upon the diagnosis of RuV-associated granulomas, it is imperative to consider the possibility of underlying immune deficiencies. Live rubella vaccines are contraindicated for individuals who have, or are potentially at risk of developing, severe antibody deficiencies, T-cell deficiencies, or innate immune defects (15).

## 4 Conclusion

In this current report, we present the occurrence of rubella virus-associated cutaneous granulomas in patients with TAP1 deficiency, which stems from a novel genetic alteration that has not been previously reported. We offer insights and recommendations pertaining to the diagnostic approach for such cases. The intriguing question of whether granulomas observed in patients with TAP1 deficiency serve as a compelling long-term reservoir for persistent RuV infection or if RuV infections contribute to the initiation and development of granulomas warrants further in-depth investigation.

## Data availability statement

The original contributions presented in the study are included in the article/**Supplementary Materials**, further inquiries can be directed to the corresponding author.

## Ethics statement

The studies involving humans were approved by The Medical Ethics Committee of the First Affiliated Hospital, Sun Yat-sen University. The studies were conducted in accordance with the local legislation and institutional requirements. The participants provided their written informed consent to participate in this study. Written informed consent was obtained from the individual(s) for the publication of any potentially identifiable images or data included in this article.

## Author contributions

QW: Data curation, Formal analysis, Investigation, Methodology, Writing – original draft, Writing – review & editing. HS: Data curation, Formal analysis, Investigation, Methodology, Project administration, Supervision, Writing – original draft, Writing – review & editing. JH: Conceptualization, Data curation, Methodology, Project administration, Resources, Supervision, Validation, Writing – review & editing. JY: Data curation, Formal analysis, Methodology, Software, Writing – review & editing. NL: Data curation, Formal analysis, Methodology, Project administration, Resources, Supervision, Writing – review & editing.
