# Supplementary figures and images for: Case report: Rubella virus-associated cutaneous granuloma in an adult with TAP1 deficiency
Source: Front Immunol. 2024 Apr 12;15:1366840. doi: 10.3389/fimmu.2024.1366840 (PMC11045939; doi:10.3389/fimmu.2024.1366840)

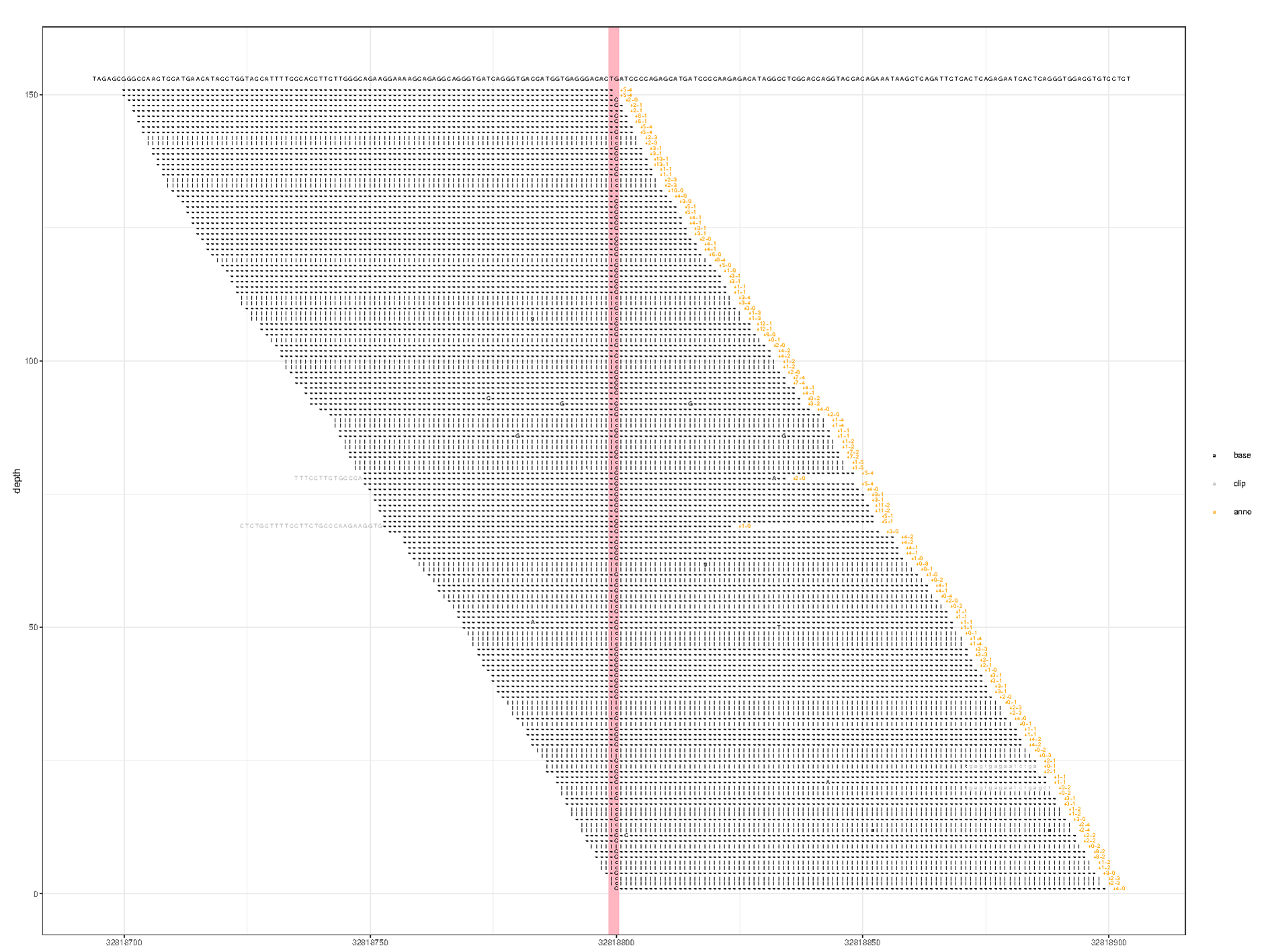

Supplement: Supplementary file 1 [file Image_1.tif]
